# THEORY-DRIVEN ROLE MODEL STORIES’ EFFECTIVENESS IN IMPROVING PALLIATIVE CARE KNOWLEDGE AMONG A DIVERSE POPULATION

**DOI:** 10.1093/geroni/igz038.2539

**Published:** 2019-11-08

**Authors:** Deborah Hoe, Anna Rahman, Kayla Johari, Susan Enguidanos

**Affiliations:** University of Southern California, Los Angeles, California, United States

## Abstract

Palliative care, specialized medical care for seriously ill people, has been demonstrated to reduce pain and symptoms while increasing satisfaction with health care. Yet, national surveys show that less than 10% of people are aware of palliative care, and among these, many believe it to be synonymous with hospice. The purpose of this study is to determine the effectiveness of 3-minute long theoretically-driven role model video stories at improving knowledge of palliative care. We recruited 161 adults age 50 and older from senior centers, assisted living, and other community-based sites. Pretest-posttest study design was employed and each participant completed 20 question surveys about palliative care knowledge, intent to seek palliative care, and perceptions about the videos. We also recorded participants’ opinions of the videos. Regression analysis was conducted to test the effectiveness of the role model video stories and qualitative analysis to elicit the major themes in participants’ opinions. Overall, palliative care knowledge score (max=13) improved from an average of 4.64 to 9.99 (t=11.99, p<0.001). Two-sample t-test revealed no significant difference in change of score by race. Regression analysis revealed that belief in the role models as real people and participants with 12 years of education significantly predicted higher change in knowledge scores. Conversely, being non-white and widowed were significant predictors of lower changes in score. Qualitative analysis of participants’ perspectives revealed two major positive themes: authenticity and informativeness of videos. This study suggests that theoretically-driven role model video stories may be an effective strategy to improve palliative care knowledge.

